# A Retrospective Analysis of Dog–Dog and Dog–Human Cases of Aggression in Northern Italy

**DOI:** 10.3390/ani10091662

**Published:** 2020-09-16

**Authors:** Lorella Notari, Simona Cannas, Ylenia Agata Di Sotto, Clara Palestrini

**Affiliations:** 1 Independent Researcher, London SW11 1RB, UK; 2 Dipartimento di Medicina Veterinaria, Università degli Studi di Milano, 20122 Milano MI, Italy; simona.cannas@unimi.it (S.C.); yleniaagata.disotto@studenti.unimi.it (Y.A.D.S.); clara.palestrini@unimi.it (C.P.)

**Keywords:** canine aggression, dangerous dogs, dog breeds, dog bites

## Abstract

**Simple Summary:**

The prevention of dog bites is an important issue with multiple safety and welfare aspects. We investigated the characteristics of dog bite incidents reported to public authorities in the North of Italy with the aim of providing more data for future risk assessment and prevention tools. We found that no breeds in particular were responsible for severe or multiple bites towards either humans or dogs, but there was a trend of defensive bites towards human beings in private homes and a trend of offensive bites towards other dogs in public areas. We also found that crossbreed dogs and dogs adopted from shelters were significantly more likely to show defensive aggression towards their owners. Our findings indicate that defensive aggression towards owners is linked to fear and anxiety, and we suggest that helping owners to have a better understanding of their dogs’ welfare, behaviour and communication via educational programs is an important prevention tool.

**Abstract:**

In this survey, a caseload of aggressive dogs (*n* = 170) was analysed to increase the available information about biting dog characteristics, contribute to risk evaluation and improve bite prevention tools. All dog data were collected from questionnaires completed by veterinary behaviourists in Northern Italy. All dogs were referred to them by public authorities to be evaluated and treated due to the incidence of one or more episodes of aggression. Between the two groups of human-directed and dog-directed aggressive dogs, significant associations were found: the dog-directed aggressive group inflicted significantly more severe bites (*p* < 0.01) and offensive aggression (*p* < 0.01), whereas defensive aggression was significantly more numerous in the human-directed aggression group (*p* < 0.01) and more significantly located in private homes (*p* < 0.01). No significant associations were found between the severity of bites and one or more specific breeds in either group. The prevalence of defensive bites in private homes in human-directed aggression indicate that owners’ understanding of their dogs’ behaviour and communication is fundamental to preventing aggression, and that owner education programmes are fundamental tools to reduce aggression risk factors and prevent aggression.

## 1. Introduction

The issue of dog biting has multiple aspects, including public health problems due to the possible physical and psychological damage to the victim and the transmission of diseases, but it also has a significant impact on dog welfare. When fear is the emotion underlying the animals’ aggressive behaviour, the owners’ attempts to physically control them might make dogs afraid of them, and this might, in turn, cause anxiety and disruption in their relationship. Furthermore, the necessity to avoid further incidents is likely to result in less exercise and social contact for dogs and, in some cases, even rehoming or euthanasia are considered. Another consequence that is deeply detrimental for dogs’ welfare is that biting dogs can be seized and confined in kennels as a consequence of the severity of the bites or the inability of their owners to properly manage and control them [1,2,3,4].

The role of genetics when discussing “dangerous dogs” is one of the most debatable areas, and although experts in this field have a more scientific perspective in terms of the genetic influence on dog behaviour, many European countries have introduced “breed lists” of some sort [5,6]. In the United Kingdom (UK), the Dangerous Dogs Act was introduced in 1991 and then amended in 1997 to prohibit four dog “types”: Pit Bull, Japanese Tosa, Dogo Argentino and Fila Brasileiro, wherein the term “type” was introduced to determine the breed of the dog from physical characteristics rather than from legal documents. Owning these dogs is illegal in the UK unless it is demonstrated that they are not dangerous and can be safely managed; following a legal process, prohibited dogs can be included in the list of exempted dogs. The owners of these dogs must be insured and keep their dogs muzzled and on a lead in public places [7]. In Germany, Breed Specific Legislation (BSL) varies across different states but the import and trade of Pit Bulls is prohibited by German Federal Law. In France, there are two categories of dogs; category one, called “Attack dogs” (Staffordshire Terrier, Pit Bull Terriers, Boerbull and Tosa Inu), is composed of dogs without pedigree papers, and all dogs in this category must be neutered. Dogs in category two are called “Guard dogs” (Staffordshire Bull Terrier, Tosa Inu and Rottweiler) and must have pedigree papers, but neutering is not compulsory. Dogs belongings to these two categories can only be owned after obtaining a licence that owners must keep with them at all times when they are with their dogs. To obtain a licence to own a dog from categories one and two, the owner must be insured and the dog must be assessed by a vet and follow an approved training course. In Spain, dog ownership is regulated by different laws, and many breeds have been identified with the potential to be dangerous. Veterinarians, following episodes of aggression, can also declare that the dog is dangerous. The owners of dangerous dogs must certify annually that they are physically and psychologically able to care for their dogs [8,9]. In Italy, the first piece of legislation was introduced back in 2003 by the Minister of Health to protect public safety after several episodes of severe dog bites which received considerable media coverage. The first version of this legislation introduced several restrictive rules for Pit Bulls and all the breeds belonging to the first and second group of the Federation Cynologique International (FCI) [10]. All dogs belonging to the breeds mentioned in the first version of the legislation had to be muzzled and kept on a lead in public places [11]. The number of breeds was then reduced to 17 in 2005, and in 2009 the list of breeds was definitively removed, and Italy adopted legislation that mainly addressed the incidents of aggression in individual dogs.

Now, as a result of dog biting or reports of episodes of aggression to the official authorities, Italian veterinary services have begun a targeted training programme for the assessment of the psychological conditions of the animal. The current legislation is a temporary law renewed year on year [12], and official veterinarians use both compulsory and discretionary criteria to refer the case to a veterinary behaviourist. Compulsory criteria are referred to the report of severe extreme bites that required medical attention with a predicted recovery period of more than 20 days, while discretionary criteria include the consideration of several risk factors such as the strength of the dog, previous episodes of aggression with or without bite, the ability of the owner to safely manage the dog, the safety of the environment and the presence of children or other fragile individuals.

The owner an of aggressive dog must have their dog undergo a compulsory veterinary behavioural examination by a certified veterinary professional recognised as an expert in behaviour medicine by the Italian Federation of Veterinarians (FNOVI) [13] and must attend a course where they learn their duties as owners and citizens and receive information about dog behaviour and correct dog management. They must also always fit the dog, when in urban areas and in places open to the public, with both a leash and muzzle.

There are no official data so far regarding the success of this kind of approach compared to the breed lists adopted in the past in Italy and currently active in other European countries. Reports regarding the efficacy of legislation based on the list of banned dog breeds or restrictions on the possession of particular breeds or types of dogs have, so far, been controversial [5,11,14]. The issue of risk evaluation appears to be extremely complex and has two main general aspects: the first is the possibility of predicting risk factors for dog bites in the general dog population, and the second is how to evaluate and predict future risks to the public of dogs that have already bitten people [15,16].

The retrospective analysis of cases presented in this paper investigates the possible associations between the characteristics of biting dogs reported as subjects posing a risk to public safety by public authorities, the characteristics of the victims, the features of aggressive episodes and the dogs’ management. The features of the episodes of aggression towards human beings and towards other dogs are analysed. This study aims to contribute to a more thorough understanding of risk factors in dog bites in order to improve prevention tools.

## 2. Materials and Methods

### 2.1. Data Collection and Analysis

Between June and September 2018, a group of veterinarians, recognised as experts in animal behaviour medicine by the FNOVI, were asked to fill in a questionnaire with data from cases referred to them for a compulsory veterinary behaviour visit following episodes of dog aggression reported to the official authorities. A request was sent to all the veterinary surgeon experts in behaviour present in the FNOVI website list and the present data came from the ones that sent filled questionnaires by the end of September 2018. They were requested to report data about the cases that were referred to them by public authorities (official veterinarians) in the past three years. The respondents were informed that the purpose of the questionnaire was to gather information about biting dogs and bite episodes.

Bite episodes, owners’ and dogs’ details can be reported to official veterinarians by law enforcement, and it is also compulsory for hospitals and physicians to report any bite lesion and the details of the dog and the dog’s owner as reported by the victim. Private veterinarians must also report to official veterinarians any bite episode they become aware of and members of the public can report episodes of bites to law enforcement. The initial evaluation of dogs and the decision to make it compulsory for their owners to undergo the behaviour visit was made by official veterinarians on the basis of the presence of one or more of the following risk factors:

1. The bite severity and a history of multiple bites;
2. The bite context, whereby even mild bites might be considered at risk for future public safety in certain circumstances (for example possibility to escape or a lack of awareness of the owner of the level of risk related to poor management);
3. Concern about the welfare of the dog;
4. Dog physical and behavioural characteristics;
5. Presence or risk of frequent interactions with possible victims who belong to vulnerable groups (e.g., elderly people, small children, disabled people).

Veterinary behaviourists were asked to complete an Excel form reporting information from the behaviour visits about the sex, age, breed, age of adoption and origin of the dogs; characteristics of the victims; bite characteristics; dog management; aggression contexts (i.e., private homes or public spaces) (see Table 1); and characteristics of aggression (see Table 2).

Breed identifications and origin of the dogs were stated by their owners and reported in this study according to the FCI classification [10].

In Housing, the information concerned whether the dog was kept indoors with or without outdoor access or kept completely outdoors; the time dogs were left alone for was divided into three time frames—less than four hours per day, between five and six hours and more than seven consecutive hours. The level of exercise was also investigated and reported as either two or more walks per day, one walk per day, irregular walks and the dog was never walked outside the property. In Training History, the information requested concerned whether the dog had a history of training with professional trainers, either in single/private courses or group courses, regardless of the method of training.

Aggression was defined as defensive when displayed to interrupt physical interactions or approaches and more likely motivated by fear or discomfort, whereas aggression was defined as offensive when the aggressor proactively attacked the victim. In dog–dog aggression, aggression was defined as defensive when the dog responded to a clear approach by another dog, for example, a dog with a neutral behaviour is chased by another dog and reacts aggressively when the chasing dog reaches them. When the aggression was perpetrated by more than one dog, this was considered as group aggression.

### 2.2. Statistical Analysis

Analysis was performed using IBM SPSS Statistics 21 with summary descriptive statistics calculated initially.

The Pearson’s Chi Square test with Bonferroni correction was used to investigate possible associations between the characteristics and management of dogs in the total sample of dogs and within each group of dogs’ aggressive behaviour towards humans and dogs’ aggressive behaviour towards other dogs, the aggression features and contexts and the victims’ and dogs’ characteristics. The ages of dogs in the two groups (dog–dog and dog–human aggressive dogs) were compared using the Mann–Whitney U test. Differences in the severity of bites in the different group ages were investigated with a one-way multivariate analysis of variance (one-way MANOVA).

## 3. Results

The files of 170 dogs presented to 11 veterinary behaviourists between January 2016 and September 2018 as a consequence of episodes of aggression that were considered as potentially dangerous for public safety were collected and analysed. The investigated sample was composed of 41.2% (*n* = 70) dogs aggressive towards other dogs, 51.2% (*n* = 87) dogs aggressive towards humans and 7.6% (*n* = 13) dogs aggressive towards other animals (cats and livestock).

### 3.1. Gender, Age and Breed

All dogs in the present sample were medium or large size. The crossbreed dog weight varied between 20 kg and 38 kg.

#### 3.1.1. Gender, Age and Breed of Dog Aggressive towards Other Dogs

Table 3 and Table 4 illustrate the gender and age of dogs aggressive towards other dogs, and the breed distribution of dogs aggressive towards other dogs is shown on Table 5.

#### 3.1.2. Gender, Age and Breed of Dogs Aggressive towards Humans

The gender and age of dogs aggressive towards humans are shown in Table 6 and Table 7 and the breed distribution and FCI group of dogs aggressive towards humans are shown in Table 8.

### 3.2. Age of Adoption and Origin

Age of adoption referred to the age when the dogs were placed in the possession of their current owners, and the origin referred to whether the dog was adopted from a professional breeder, a private family or a shelter.

#### 3.2.1. Age of Adoption and Origin of Dogs Aggressive towards Other Dogs

In the group of dogs aggressive towards other dogs, 15.7% (*n* = 11) had been in their owners’ possession since they were less than two months old; 48.6% (*n* = 34) had been in their owners’ possession since they were between two and three months old; 20% (*n* = 14) had been in their owners’ possession since they were between four and twelve months old and 15.7% (*n* = 11) came into their owners’ possession when they were older than one year of age. The origins of the dogs are illustrated in Table 9.

#### 3.2.2. Age of Adoption and Origin of Dogs Aggressive towards Humans

In the group of dogs aggressive towards humans, 10.3% (*n* = 9) had been in their owners’ possession since they were less than two months old; 60.9% (*n* = 53) had been in their owners’ possession since they were between two and three months old; 9.2% *n* = 8) had been in their owners’ possession since when they were between four and 12 months old; and 17.2% (*n* = 15) came into their owners’ possession when they were older than one year of age. For 2.3% (*n* = 2) dogs, this information was not available. The origins of dogs aggressive towards humans are illustrated in Table 10.

### 3.3. Dog Management

Dog management refers to whether the dogs had a previous history of training, how they were housed, how long they were regularly left alone in an average day, and how many times per day they were walked.

#### 3.3.1. Management of Dogs Aggressive towards Other Dogs

In the dog–dog aggression group, history of previous training was available for 69 dogs (98.6%). Twenty-one (30%) dogs had a history of training. Housing information was available for 68 dogs (97.1%) dogs. Twenty-one dogs (30%) were housed indoors only, 32 dogs (45.7%) were housed indoors with free access to a garden, 12 dogs were housed exclusively in the owner’s garden (17.1%), and two dogs (2.9%) were housed in an outdoor kennel. Information regarding how long the dog was regularly left alone on an average day was available for 43 dogs in this group (61.4%). Twenty-three (32.9%) were left alone for less than four hours, 12 (17.1%) were left alone for five–six hours and eight (11.4%) were left alone for more than seven hours. Information regarding daily exercise was available for 63 dogs (90%). Forty-three (61.4%) were walked more than two times per day, two (2.9%) were walked once per day, five (7.1%) were walked in an irregular way (not every day) and 13 (18.6%) were never walked.

#### 3.3.2. Management of Dogs Aggressive towards Humans

In the dog–human aggression group, history of previous training was available for 86 dogs (98.5%). Thirty (34.5%) dogs had a history of training. Housing information was available for 85 dogs (97.6%) dogs. Twenty-seven (31%) were housed indoors only, 37 (42.5%) were housed indoors with free access to a garden, 20 were housed exclusively in the owner’s garden (23%) and only one dog (1.1%) was housed in an outdoor kennel. For two dogs, the information was not available (2.3%). Information regarding how long the dog was regularly left alone on an average day was available for 68 dogs in this group (78.1%). Forty-nine (56.3%) were left alone for less than four hours, 9 (10.3%) were left alone for five–six hours and ten (11.5%) were left alone for more than seven hours. For 19 dogs (21.8%), this information was not available. Furthermore, information regarding daily exercise was available for 76 dogs (87.4%). Thirty-eight (43.7%) were walked more than two times per day, ten (11.5%) were walked once per day, 18 (20.7%) were walked in an irregular way (not every day) and 10 (11.5%) were never walked. This information was not available for 11 dogs (12.6%).

### 3.4. Aggression Features

Aggression features referred to the location where the aggression occurred, the severity of bites and whether the aggression was considered offensive or defensive.

#### 3.4.1. Aggression Features of Dogs Aggressive towards Other Dogs

The aggressive episodes in this group took place in private homes in 11 cases (15.7%) and in public spaces including dog areas in 59 cases (84.3%). Regarding severity of bites, mild bites totalled 17 cases (24.3%), average severe bites 11 (15.7%), severe bites five (7.1%) and in 30 cases they caused the death of the victim (42.9%). For seven cases of dog–dog aggression (10%), the information about the severity of bites was not available. In this group, 16 dogs (22.9%) had a history of previous aggressive episodes with bites. Sixty-three dogs (90%) were offensively aggressive, five dogs (7.1%) were defensively aggressive, and for two dogs (2.9%) the information about the type of aggression was not available. Seventeen dogs (24.3%) were involved in group aggression and all group aggression was directed towards strange dogs.

#### 3.4.2. Aggression Features of Dogs Aggressive towards Humans

In the dog–human aggression group, an unfamiliar human was the victim in 35 cases (40.2%), a known adult human being/family member the victim in 16 cases (18.4%) and the owner the victim in 13 cases (14.9%). Non-family children were the victims in eight cases (9.2%), whereas family children were the victims in seven cases (8%). In eight cases (9.2%) the victim was not clearly specified. The aggression episodes took place in private homes in 54 cases (62.1%) and in public spaces including dog areas in 33 cases (37.9%).

In bites directed towards humans, 58 (66.7%) were on the arms or legs, 13 (14.9%) on the face, head or neck, one (1.1%) was on the back of the victim, one (1.1%) on the thorax, one on the abdomen (1.1%) and seven on multiple sites (8%). In six cases (6.9%), the information about the bite site in humans was not available. In this group mild bites totalled 39 (44.8%), average severe bites 23 (26.4%), severe bites 18 (20.7%) and in three cases (3.4%) the bite caused permanent physical injuries. For four cases (5.7%), the information about the severity of bites was not available. Thirty-nine dogs aggressive towards humans (44.8%) had a history of previous aggressive episodes with bites, and one dog (1.1%) had a history of previous aggressive episodes but no bites. Twenty-seven dogs (31.0%) were offensively aggressive, 54 (62.1%) were defensively aggressive, and for six dogs (6.9%) the information about the type of aggression was not available. No group aggression was reported in the group of dogs aggressive towards humans.

### 3.5. Within Group Correlations

A Pearson Chi-Square with Bonferroni correction test revealed significant associations in the two groups of dogs aggressive towards other dogs and dogs aggressive towards humans.

#### 3.5.1. Correlation in the Group of Dogs Aggressive towards Other Dogs

The gender of dogs and multiple aggression (history of previous episodes of aggression) were significantly associated (*χ*^2^ = 17.331, *p* < 0.01). The number of neutered females was significantly higher than expected (Table 11). The gender of dogs was also associated with group aggression: females were responsible for significantly more group aggression (*χ*^2^ = 8.996, *p* < 0.05). Housing in multi-dog households was significantly correlated with aggression incident location: dogs in multi-dog households inflicted significantly more bites in the owner’s property (*χ*^2^ = 6.631, *p* < 0.05) compared with dogs in single-dog households. Daily exercise was also correlated with group aggression: dogs that were never walked were responsible for significantly more group aggression (*χ*^2^ = 25.651, *p* < 0.01). The comparisons between dog daily exercise (number of walks per day) and group aggression episodes are reported in Table 12. For seven dogs, the information about daily exercise was not available.

#### 3.5.2. Correlation in the Group of Dogs Aggressive towards Humans

Significant associations were found between type of aggression (i.e., defensive or offensive) and breed group: mongrel and crossbreeds were responsible for significantly more defensive aggression (*χ*^2^ = 15.831, *p* < 0.05). Comparisons of types of aggression in the different FCI breed groups, Pit Bulls and mongrels are shown in Table 13. Significant associations were also found between the type of aggression and the origin of dogs: dogs adopted from private owners and shelters were responsible for significantly more defensive bites, whereas dogs adopted from breeders were responsible for significantly more offensive bites (*χ*^2^ = 14.825, *p* < 0.01). Comparisons of types of aggression in dogs adopted from breeders, private owners, shelters or other origins are shown in Table 14. Furthermore, significant associations were found between breed group and type of victim (*χ*^2^ = 36.508, *p* < 0.05): mongrels and crossbreeds were responsible for significantly more bites towards adult family members. The comparisons between breed group and type of victim are shown in Table 15. Significant associations were also found between dog origin and the type of victim: dogs adopted from shelters were responsible for significantly more bites directed towards adults of their family, whereas dogs adopted from breeders were responsible for significantly more bites directed towards strangers (*χ*^2^ = 18.008, *p* < 0.05). The comparisons between the origin of dogs and the types of victim are shown in Table 16.

Age of adoption and bite severity were also significantly associated: dogs adopted when they were more than one year old were responsible for significantly more severe bites that required stitches with over 20 days of prognosis (*χ*^2^ = 26.088, *p* < 0.01). The comparisons between age of adoption and severity of bites are shown in Table 17. Significantly more bites towards children were located on the face, head and neck, whereas in adults they were located more on the legs and arms (*χ*^2^ = 51.718, *p* < 0.01). Table 18 reports the comparisons between type of victim and bite site. 

### 3.6. Between Group Analysis

Considering both groups (dog–dog and dog–human aggression), significant associations were found between the type of victim (human or dog) and the type of aggression (*χ*^2^ = 58.028, *p* < 0.01). The comparisons between types of aggression in the dogs aggressive towards human beings (*n* = 87) and dogs aggressive towards other dogs (*n* = 70) are shown in Table 19. Significant associations were found between the severity of bites and the type of victim (χ^2^ = 52.081, *p* < 0.01): the comparisons between the severity of bites in the dogs aggressive towards human beings (*n* = 83) and dogs aggressive towards other dogs (*n* = 63) are shown in Table 20. Furthermore, significant associations were found between the type of victim (dog or human being) and history of multiple episodes of aggression (*χ*^2^ = 15.475, *p* < 0.05): the comparisons between the history of previous aggressive episodes in the dogs aggressive towards human beings (*n* = 85) and dogs aggressive towards other dogs (*n* = 70) are shown in Table 21. Significant associations were also found between the type of victim (dog or human being) and the incident location (*χ*^2^ = 36.134, *p* < 0.01): the comparisons between locations of bite incidents in the dog aggressive towards human beings (*n* = 87) and dogs aggressive towards other dogs (*n* = 70) are shown in Table 22. Furthermore, significant associations were found between the type of victim (dog or human being) and multi-dog households: dogs were more significantly the victims of aggression perpetrated by dogs that lived in a multi-dog households (*χ*^2^ = 11.487, *p* < 0.01); in Table 23 the comparisons between dogs housed in single- or multi-dog households and the type of victim, human or dog, are illustrated. Significant associations were also found between the type of victim (dog or human being) and dog gender (*χ*^2^ = 9.080, *p* < 0.05); in Table 24 the comparisons between dog gender and the type of victim, human or dog are illustrated.

The mean ages of dogs in the two groups were not normally distributed; to compare them, the non-parametric Mann–Whitney U test was used. A significant difference in ages between the two groups was found: dogs aggressive towards humans were significantly younger than dogs aggressive towards other dogs (Mann-Whitney U = 2421, *p* < 0.05). One-way MANOVA showed no statistically significant difference in the severity of bites in the different age groups.

## 4. Discussion

In this retrospective study, all the data were provided by certified veterinary behaviourists who directly carried out consultations with these dogs and their owners. The method of collecting data provided from reliable sources—veterinary behaviourists—was due to the specifics of the research objectives. Data provided by experts allowed for information to be obtained from an informed perspective [17,18,19]. Although some information might be considered more as opinion than objective data, expert opinions should be accepted as reliable and reasonably objective [20].

The first relevant piece of information obtained from the whole data set concerned breed and sex characteristics. Breeds belonging to FCI 1, 2 and 3 were largely represented and together constituted 57.1% of the entire sample. Pit Bulls were also largely represented (12.4%). It is interesting to compare the data regarding breeds in the present sample with the general Italian dog population. Data concerning gender and breed distribution were requested to and kindly made available by the official veterinarian responsible for the canine registry office of the National Health Minister, up to the date of 9 January 2019.

When compared to the distribution of the same breeds in the general dog population, dogs belonging to group 1, 2 and 3 of the FCI in the sample of biting dogs considered in this survey were more numerous than expected. The frequency of German Shepherds in the sample of this study was 11.8%, whereas its frequency in the general dog population is 5.51%. The frequency of Rottweilers in our sample was 7.6%, whereas the frequency in the general dog population was 1.13%. In addition, there were quite a high number of Argentinian Dogos in our sample (*n*. = 10); the frequency of this breed was 5.9%, whereas it is 1% in the general dog population (no precise numbers were provided for breeds with less than 1% frequency in the general dog population). Regarding the breeds belonging to FCI group 3 (terrier), the frequency of American Staffordshire Terriers in our sample was 9.4%, whereas in the general dog population it is less than 1%. In contrast, the percentage of crossbreed and mongrel dogs in the sample of biting dogs reported here (23.5%) was lower than the reported percentage in the general Italian dog population (36.5%). There were some breeds, such as Boxers and Labradors, whose frequency in the present study more or less matched the frequency in the general dog population. It was difficult to compare the general population of Pit Bulls and other dog breeds not recognised by the FCI to the National database, because in some Italian Regions they were classified as “Breeds not belonging to FCI”, whereas in other Regions they were classified as “Pit Bulls”. Meanwhile, crossbreed and mongrels in some Italian Regions were classified as “Breed not belonging to FCI”, and in some other Regions they were classified as “Crossbreed”. The National data are therefore confusing in this respect, because in “Breed not belonging to FCI”, a number of crossbreed and mongrel dogs might be included, along with Pit Bulls and other non-recognised breeds. This means that it is currently extremely difficult to understand the real distribution of one of the more debated breeds/types. Furthermore, it is most likely not possible to make a distinction between Pit Bulls and American Staffordshire Terrier due to the absence of a pedigree document [21,22]. In this survey, breed recognition was based on the national canine registry which is, in turn, based on the owners’ statements and dogs’ appearance. This criteria are probably not very accurate in general; a recent study showed that the ability to recognise banned types of dogs in UK was very poor [23].

To what extent the breed is a predisposing factor for aggression is still widely debated [6,24], and a number of elements should be considered when data such as that presented here seem to demonstrate that some breeds are more likely to display aggression than others. The low number of dogs included in this database is a limitation in this study, and it must be considered that aggressive episodes done by dogs of other breeds might have been less likely to be reported to authorities because of different personal perceptions of dangerousness [25].

In the present study, more males were reported for episodes of aggression compared to females. This aspect has been demonstrated in several studies, and it might be explained by the tendency of males to be more competitive and more prone to use confrontation rather than appeasement in social conflict [26,27]. A significant difference in gender distribution was found between the group of dogs aggressive towards humans and the group of dogs aggressive towards other dogs, with a higher number of entire and spayed females in the latter group. In an earlier study, a prevalence of females was found in cases of intraspecific aggression [28]. Within the group of dogs aggressive towards other dogs, females and neutered females were also responsible for significantly more multiple aggression and group aggression. It is worth reflecting on the common belief that gonadectomy decreases the risk of aggression, a belief that does not seem to be supported by evidence [29,30]. This particular issue is still being debated and correlated with information about the age of neutering and the dogs’ behaviour before they were neutered, as the reason for the neutering might have been that they were aggressive.

Intraspecific aggression was mostly offensive and ended in the death of the victims in 30 cases. In the dogs aggressive towards other dog groups, aggression was associated with being housed with other dogs (a multi-dog household) and a low level of exercise (less than one walk per day or no walk at all). These findings appear to indicate that owner management is an important element of prevention. The group aggression reported here was all directed towards strange dogs: most likely, intraspecific aggression among dogs that live in the same household is less likely to be reported to authorities.

In human-directed aggression, there was a prevalence of defensive aggression, and crossbreed dogs showed more defensive aggression than pure breed dogs and Pit Bulls. The definition of defensive or offensive aggression presented here was fairly simplified and was consistent with the identification of types of aggression reported by Frank [31] for the sake of the interpretation of the aggression contexts; furthermore, some aggression classified as “offensive” might have been motivated instead by social conflict. In intraspecific aggression episodes, the prevalence of offensive bites was probably related to the fact that dogs that proactively attacked other dogs were more likely to be reported to public authorities than dogs that reacted to a conspecific’s attack. The majority of aggression towards human beings was classified as defensive, defined as an act displayed to interrupt physical interactions or approaches, suggesting that improving the owners’ ability to both predict their dogs’ behaviours and interpret body language signals might have reduced the risk of this kind of aggression [32,33].

The importance of the dog–owner relationship and communication seem further supported by the finding that in human-directed aggression, the majority of aggressive episodes took place in private homes and were defensive. Other authors have reported that the most common context of a dog bite is related to approaches to the animal and attempts to physically interact with it, and our findings seems to confirm this [2]. Dogs adopted from shelters inflicted significantly more defensive bites towards their owners, whereas dogs adopted from breeders inflicted significantly more offensive bites towards strangers, and dogs adopted when they were more than one year old inflicted more severe bites. Several studies have reported that fearfulness is one of the most reported problems in dogs adopted from shelters, and adoption from shelter as well as adoption at a later age may be related with insufficient early socialisation and training. It has been shown that fearful dogs had fewer early life experiences, and this was also associated with a prevalence of anxiety later in life. We might hypothesise that dogs adopted at a later age and dogs adopted from shelters are more likely to be more prone to defensive aggression because they are more prone to be fearful and to suffer from anxiety-related disorders [34,35,36]. It should also be considered that behaviour problems, including aggression towards people or other dogs, have been reported to be frequent reasons for dog relinquishment in shelters [37]. A significant association was found between aggression towards humans and history of multiple aggressive episodes, and this may be because aggression towards humans is more likely to be reported to authorities, also considering that emergency hospital services and physicians have the duty to report reasons for injuries.

Within the human aggressive dog group, a significant association was found between the type of human victim and the sites of bite injuries: adult humans were more likely to be bitten on their arms or legs, whereas children were significantly more likely to be bitten on their head, face or neck. These findings have been reported in several studies [38,39] and might be explained by the small size of children, whose faces are at the dog muzzle level and by their limited ability to detect even the clearer signals of an incoming aggression such as a growl. Children can unknowingly provoke dogs with their natural tendencies to be active and loud, and parent supervision is always of paramount importance to prevent bites [40,41]. Dogs can be important in children’s lives in many ways because they provide enjoyment and help children develop responsibilities but, especially for families with young children, parents should have a good understanding of dog communication, behaviour and welfare to minimise any risk of aggression [42,43].

In our sample, the dogs in the dog–human aggressive groups were significantly younger than the dogs in the dog–dog aggressive group, but no differences in the severity of bites in the different group ages were found. In our sample, only five dogs were less than one year old, and it is difficult to state whether “puppy bites” were different from adult bites. Four of these very young dogs, two American Staffordshire (eight and ten months old), one Pit Bull (nine months old) and one Anatolian Karabash (ten months old) bit human beings; one Rottweiler (nine months old) bit and killed another dog in a public area. The Anatolian Karabash inflicted a severe bite on a family member that approached him while he was on lead with his owner, one Pit Bull inflicted a mild defensive bite on a stranger approaching him in his owner’s property and the other two dogs inflicted mild offensive bites on strangers in public areas (one adult and one child). These two latter incidents were interpreted by the dogs’ owners as “attempts to play” but perceived by the victims as proper attacks; it is most likely that this perception (and the report to the public authorities of the two mild incidents) was influenced by the dogs’ physical appearance.

Other authors have reported that aggression towards other dogs was associated with increasing dog age, but it has also been reported that older dogs were more prone to be aggressive towards unfamiliar people entering the house and more aggressive towards their owners [44,45,46]. Our findings are related to a population of biting dogs reported to the authorities; we did not compare the age of dogs in our sample with a “normal” dog population.

## 5. Conclusions

The episodes of dog–dog and dog–human aggression have some different features in terms of where the aggression takes place, the severity of bites, if it was an offensive or defensive aggression, and the dogs’ characteristics. Elements related to early experiences, origins and the management of dogs in both intra- and interspecific aggression seem to play an important role. We found several associations between the characteristics of dogs, their management and the type of aggression, but no associations between individual breeds and the severity of bites or repeated aggressive episodes. The over-representation of some breed groups might have been related to diffuse stereotyped perceptions which increased the probability of public complaints and reports to public authorities about some breeds. An important finding that will contribute to shaping effective prevention strategies is that most of the human-directed aggression that occurred in private homes was defensive, indicating that owners’ understanding of their dogs’ behaviour and communication are fundamental to preventing such aggression. The results in this retrospective study suggest that educational programmes for owners are fundamental tools to reduce aggression risk factors and prevent aggression. The relatively small sample size of this survey is an important limitation for our conclusions and further research with a larger sample of dogs is necessary to provide more information about risk factors for dog bites.

## Figures and Tables

**Table 1:** Summary of the investigated areas.

Dog Characteristics	Victim Characteristics	Aggression Features	Dog Management	Aggression Contexts
Gender	Owner	Severity of Bites	Housing	Private Home or Garden
Age	Family Member/Friend	Localisation of Bites	Home Management	Public Spaces
Breed	Stranger	History of Previous Aggressive Episodes	Daily Exercise	Dog Areas (fenced public spaces where dogs can be off lead)
Age of Adoption	Child of the Family	Defensive or Offensive Aggression	Training History	
Origin (Breeder, Shelter, Other)	Stranger Child	Group Aggression		
	Dog			
	Cats or Other Animals			

**Table 2:** Descriptions of severity of bites, site of bites in human victims and history of multiple aggressive episodes.

Description of Damage Caused by Bites	Localisation of Bites in Human Victims	Multiple Aggression
Mild injury, no stitches	Arms or legs	No previous episodes of aggression reported
Injury of average severity with a prognosis of less than 20 days	Face/head/neck	Previous episodes of aggression that did not result in bites reported
Severe injury with prognosis over 20 days	Thorax	Previous episodes of aggression with bites reported
Very severe injury with possible long-term/permanent disability	Abdomen	
Death of the victim	Multiple sites	

**Table 3:** Gender of dogs aggressive towards other dogs.

Gender	Frequency (*n*)	Percent (%)
Male	42	60.0
Female	16	22.9
Neutered male	8	11.4
Neutered female	4	5.7
Total	70	100.0

**Table 4:** Age of dogs aggressive towards other dogs.

Age	Frequency (*n*)	Percent (%)
0–11 months	1	1.4
1–2 years	14	20.0
3–4 years	21	30.0
5–9 years	28	40.0
More than 9 years	6	8.6
Total	70	100.0

**Table 5:** Breed distribution and correspondent Federation Cynologique International (FCI) group of dogs aggressive towards other dogs.

Dog Breed	Frequency (*n*)	Percent (%)	FCI Group
German Shepherd	6	8.6	1
Czechoslovakian Wolf Dog	1	1.4	1
Belgian Shepherd	1	1.4	1
Malinois	1	1.4	1
Maremmano Abruzzese Shepherd	1	1.4	1
Rottweiler	7	10.0	2
Argentinian Dogo	7	10.0	2
Dog de Bordeaux	1	1.4	2
Central Asian Mastiff	1	1.4	2
Bull Mastiff	1	1.4	2
Newfoundland	1	1.4	2
Bull Terrier	4	5.7	3
American Staffordshire Terrier	8	11.4	3
American Akita	1	1.4	5
Siberian Husky	1	1.4	5
Labrador Retriever	3	4.3	8
Greyhound	2	2.9	10
Pit Bull and American Pit Bull	11	15.7	n/a
Crossbreed and mongrels	12	17.1	
Total	70	100.0	

**Table 6:** Gender of dogs aggressive towards humans.

Dog Gender	Frequency (*n*)	Percent %
Male	69	79.3
Female	7	8.0
Neutered male	9	10.3
Neutered female	2	2.3
Total	87	100.0

**Table 7:** Ages of dogs aggressive towards humans.

Dog Age	Frequency (*n*)	Percent %
0–11 months	4	4.6
1–2 years	30	34.5
3–4 years	22	25.3
5–9 years	27	31.0
More than 9 years	4	4.6
Total	87	100.0

**Table 8:** Breed and FCI group of dogs aggressive towards human beings.

Dog Breed	Frequency (*n*)	Percent (%)	FCI Group
German Shepherd	11	12.6	1
Czechoslovakian Wolf Dog	2	2.3	1
Maremmano Abruzzese Shepherd	2	2.3	1
Belgian Shepherd	1	1.1	1
Australian Shepherd	1	1.1	1
Switzerland Shepherd	1	1.1	1
Rottweiler	6	6.9	2
Argentinian Dogo	3	3.4	2
Corso Dog	2	2.3	2
Doberman	2	2.3	2
Boxer	2	2.3	2
Dog de Bordeaux	1	1,1	2
English Bulldog	1	1.1	2
Central Asian Mastiff	1	1.1	2
Bernese	1	1.1	2
Great Dane	1	1.1	2
Neapolitan Mastiff	1	1.1	2
St Bernard	2	2.3	2
Anatolian Karabash (Kangal)	1	1.1	2
Bull Terrier	1	1.1	3
American Staffordshire Terrier	7	8.0	3
American Akita	1	1.1	5
Akita Inu	1	1.1	5
Chow Chow	1	1.1	5
Epagneul Breton	1	1.1	7
Pit Bull and American Pit Bull	9	10.3	n/a
Crossbreed and mongrels	24	27.6	
Total	87	100.0	

**Table 9:** Origins of dogs aggressive towards other dogs.

Origins	Frequency (*n*)	Percent (%)
Breeder	25	35.7
Private owners	33	47.1
Shelter	9	12.9
Other	3	4.3
Total	70	100.0

**Table 10:** Origins of dogs aggressive towards humans.

Origins	Frequency (*n*)	Percent (%)
Breeder	28	32.2
Private owners	44	50.6
Shelter	10	11.5
Other	5	5.7
Total	87	100.0

**Table 11:** Comparison of the history of previous episodes of aggression towards other dogs in different dog genders.

Dog Gender	History of Previous Episodes of Aggression	
	Yes (*n*)	No (*n*)
Male	8	34
Female	1	15
Neutered male	3	5
Neutered female	4	0

**Table 12:** Comparison of dog daily walks and episodes of group aggression towards other dogs.

Dog Daily Walks	Group Aggression	
	Yes (*n*)	No (*n*)
More than 2 walks per day	5	38
One walk per day	0	2
Irregular walks	0	5
Never walked	10	3

**Table 13:** Comparison of the type of dog–human aggression in different breed groups.

Breed Groups	Offensive Aggression (*n*)	Defensive Aggression (*n*)
FCI 1 Sheepdogs and Cattle dogs	7	11
FCI 2 Pinscher and Schnauzer–Molossoids and Swiss Mountain and Cattle dogs	12	14
FCI 3 (Terrier)	4	3
FCI 5 (Spitz and primitive types)	1	2
FCI 7 (Pointing dog)	1	0
Pit Bulls	4	4
Mongrels and crossbreeds	1	23

**Table 14:** Comparison of the type of dog–human aggression in dogs with different origins.

Dog Origin	Offensive Aggression (*n*)	Defensive Aggression (*n*)
Breeder	17	11
Private owners	12	32
Shelter	1	9
Other	0	5

**Table 15:** Comparison of the type of victim and dog breed groups.

Breed Group	Victim			
	Adult of the Family (*n*)	Stranger Adult (*n*)	Child of the Family (*n*)	Stranger Child (*n*)
FCI 1 Sheepdogs and Cattle dogs	3	11	1	3
FCI 2 Pinscher and Schnauzer–Molossoids and Swiss Mountain and Cattle dogs	8	13	3	2
FCI 3 (Terrier)	3	3	0	1
FCI 5 (Spitz and primitive types)	1	2	0	0
FCI 7 (Pointing dog)	1	0	0	0
Pit Bulls	4	3	0	1
Mongrels and crossbreeds	14	6	3	1

**Table 16:** Comparisons between the type of victim and dog origin.

Origin	Victim			
	Adult of the Family (*n*)	Stranger Adult (*n*)	Child of the Family (*n*)	Stranger Child (*n*)
Breeders	3	19	3	3
Private owners	19	16	3	5
Shelter	7	2	1	0
Others	3	1	0	0

**Table 17:** Comparisons between dog adoption age and bite severity in dog–human aggression episodes.

Adoption Age	Bite Severity			
	Mild No Stitches (*n*)	Average with Stitches, Prognosis Less than 20 Days (*n*)	Severe with Stitches, Prognosis More than 20 Days (*n*)	Permanent Physical Injuries (*n*)
0–59 days	3	3	1	1
2–3 months	30	12	6	2
4–12 months	1	5	2	0
More than one year	5	1	9	0

**Table 18:** Comparisons between the type of victim and bite site in dog–human aggression episodes.

Bite Site	Victim			
	Adult of the Family (*n*)	Stranger Adult (*n*)	Child of the Family (*n*)	Stranger Child (*n*)
Information not available	1	3	0	1
Arms or legs	26	29	0	2
Face, head, neck	2	2	5	4
Back	0	0	0	1
Thorax	0	1	0	0
Abdomen	1	0	0	0
Multiple sites	2	3	2	0

**Table 19:** Comparisons between the type of victim and type of aggression.

Type of Aggression	Victim	
	Human (*n* = 87)	Dog (*n* = 70)
Offensive	30	65
Defensive	57	5

**Table 20:** Comparisons between the type of victim and severity of bites.

Severity of Bites	Victim	
	Human (*n* = 83)	Dog (*n* = 63)
Mild, no stitches	39	17
Average with stitches, prognosis less than 20 days	23	11
Severe with stitches, prognosis more than 20 days	18	5
Permanent physical injuries	3	0
Victim death	0	30

**Table 21:** Comparisons between the type of victim and history of previous aggressive episodes.

History of Multiple Aggressive Episodes	Victim	
	Human (*n* = 85)	Dog (*n* = 70)
Yes, with bite	39	16
Yes, but no bite	1	0
No	45	54

**Table 22:** Comparisons between bite incident locations and type of victim.

Bite Incident Location	Victim	
	Human (*n* = 87)	Dog (*n* = 70)
Owner’s property	54	11
Public areas	33	59

**Table 23:** Comparisons between dogs housed in multi- or single-dog households and the type of victim.

Type of Household	Victim	
	Human (*n* = 87)	Dog (*n* = 70)
Multi-dog household	21	35
Single-dog household	66	35

**Table 24:** Comparisons between dog gender and type of victim.

Dog Gender	Victim	
	Human (*n* = 87)	Dog (*n* = 70)
Male	69	42
Female	7	16
Neutered male	9	8
Neutered female	2	4
